# Clinical outcomes and molecular characteristics of lung-only and liver-only metastatic pancreatic cancer: results from a real-world evidence database

**DOI:** 10.1093/oncolo/oyaf007

**Published:** 2025-03-13

**Authors:** Abrahm Levi, Edik Blais, John Davelaar, Matthew I Ebia, Angela Minasyan, Nima Nikravesh, Gillian Gresham, Lei Zheng, Jennifer W Chuy, Rachna T Shroff, Raymond Couric Wadlow, Patricia DeArbeloa, Lynn McCormick Matrisian, Emmanuel Petricoin, Michael J Pishvaian, Jun Gong, Andrew Eugene Hendifar, Arsen Osipov

**Affiliations:** Cedars-Sinai Medical Center, Los Angeles, CA, United States; Perthera Inc., McLean, VA, United States; Cedars-Sinai Medical Center, Los Angeles, CA, United States; Cedars-Sinai Medical Center, Los Angeles, CA, United States; Cedars-Sinai Medical Center, Los Angeles, CA, United States; Cedars-Sinai Medical Center, Los Angeles, CA, United States; Cedars-Sinai Medical Center, Los Angeles, CA, United States; University of Texas Health Science Center San Antonio, Hematology and Oncology, San Antonio, TX, United States; NYU Langone Health, New York, NY, United States; University of Arizona College of Medicine, Hematology and Oncology, Tucson, AZ, United States; Inova, Fairfax, VA, United States; Perthera Inc., McLean, VA, United States; Pancreatic Cancer Action Network, Manhattan Beach, CA, United States; Perthera Inc., McLean, VA, United States; University of Texas Health Science Center San Antonio, Hematology and Oncology, San Antonio, TX, United States; Johns Hopkins Medicine, Sidney Kimmel Comprehensive Cancer Center, Baltimore, MD, United States; Cedars-Sinai Medical Center, Los Angeles, CA, United States; Cedars-Sinai Medical Center, Los Angeles, CA, United States; Cedars-Sinai Medical Center, Los Angeles, CA, United States

**Keywords:** pancreatic cancer, metastasis, genetic profile, prognostic factors, treatment outcome

## Abstract

**Background:**

Previous research demonstrates longer survival for patients with lung-only metastatic pancreatic adenocarcinoma (mPDAC) compared to liver-only mPDAC. The objective of this study is to understand the survival differences, impact of chemotherapy, and associated genomic features of mPDAC that is isolated to either the liver or lung.

**Patients and methods:**

Longitudinal clinical outcomes and molecular sequencing data were retrospectively analyzed across 831 patients with PDAC across all stages whose tumors first metastasized to the liver or lung. Survival differences were evaluated using Cox regression. Mutational frequency differences were evaluated using Fisher’s exact test.

**Results:**

Median overall survival (mOS) was shorter in patients with liver-only metastasis (1.3y [1.2-1.4], *n* = 689) compared to lung-only metastasis (2.1y [1.9-2.5], *n* = 142) (*P* = .000000588, HR = 2.00 [1.53-2.63]. Survival differences were observed regardless of choice of 1st-line standard-of-care therapy. For 5-fluorouracil-based therapies, mOS for liver-only mPDAC was 1.4y [1.3-1.6] (*n* = 211) compared to 2.1y [1.8-3.3] for lung-only mPDAC (*n* = 175) (*P* = .008113, HR = 1.75 [1.16-2.65]). For gemcitabine/nab-paclitaxel therapy, mOS for liver-only mPDAC was 1.2y [1.1-1.5] (*n* = 175) compared to 2.1y [1.6-3.4] for lung-only disease (*n* = 32) (*P* = .01863, HR = 1.84 [1.11-3.06]). PDAC tumors with liver-only metastases were modestly enriched (unadjustable *P* < .05) for: *TP53* mutations, *MYC* amplifications, inactivating *CDK2NA* alterations, inactivating *SMAD* alterations, and *SWI/SWF* pathway mutations. PDAC tumors with lung-only metastases were enriched for: *STK11* mutations, *CCND1* amplifications, and *GNAS* alterations.

**Conclusion:**

Patients with lung-only mPDAC demonstrate an improved prognosis relative to those with liver-only mPDAC. Responses to chemotherapy do not explain these differences. Organotropic metastatic tumor diversity is mirrored at the molecular level in PDAC.

**Implications for practice**

Previous research has shown a longer survival for patients with lung-only metastatic pancreatic adenocarcinoma (mPDAC) relative to liver-only mPDAC. However, the genomic characteristics associated with PDAC that has metastasized to either the lung- or liver-only are not well-defined. This study describes the survival differences and associated molecular features between lung- and liver-only mPDAC. Our data suggest sites of metastases as important prognostic variables and thus requires further exploration of site-specific biology in pancreatic cancer metastasis.

## Introduction

Pancreatic cancer is the third leading cause of cancer-related deaths in the US, with the lowest 5-year survival rate of 11% across all stages.^1^ In 2022, there were an estimated 62 210 new cases of pancreatic cancer and 49 830 deaths.^1^ By 2040, pancreatic cancer is estimated to be the second leading cause of cancer associated mortality.^2^ 50% of patients have metastatic disease at presentation, 30%-35% with borderline resectable or locally advanced unresectable disease, and only 10%-15% of patients with localized disease suitable for surgery; however, outcomes for unresectable or advanced stage diagnoses continue to be poor.^3,4^ Multiagent cytotoxic regimens of systemic therapies consisting of gemcitabine/nab-paclitaxel or FOLFIRINOX remain the preferred treatments for patients with metastatic pancreatic ductal adenocarcinoma (mPDAC), though these therapies have only resulted in modest clinical improvements.^3,5^

Common sites of metastases for PDAC include the liver (80-90%), lymph nodes (25%), lung (25-40%), peritoneum (25-40%), and bones (10-15%).^3,6^ Although the median overall survival (mOS) time of mPDAC is about 6-11 months, multiple investigations have reported a longer survival for patients with isolated pulmonary metastasis at diagnosis.^7-10^ Specifically, mPDAC patients with isolated pulmonary metastasis exhibit a greater mOS in comparison to those with liver-only metastasis.^8,10,11^ This suggests that lung-only mPDAC are a distinct subgroup of PDAC with improved prognosis compared to other metastatic sites of PDAC.^8-11^ It remains unclear what the biologic drivers are of lung-only mPDAC.^9,12^ Specifically, genomic characteristics associated with PDAC that has metastasized to either the lung or liver only are not well-defined. A detailed study of the genomic landscape of site-specific metastasis of PDAC may provide insight into distinct subgroups of advanced pancreatic cancer, which can have implications not only on treatment approaches and novel drug targeting, but also prognosis. The aim of this study is to understand the survival differences and associated specific molecular features of mPDAC that is isolated to either the liver or the lung only.

## Materials and methods

In this retrospective study, we performed exploratory analyses to understand potential clinical and molecular differences in patients diagnosed with mPDAC whose tumors first metastasized to either the lung or the liver. We utilized Perthera’s real-world evidence (RWE) database where patients in the US were registered on an IRB-approved observational protocol (WCG IRB Protocol ID: PCT-01-012) via physician referrals directly to Perthera or via the Know Your Tumor Program, a patient-centered initiative conducted by Perthera and the Pancreatic Cancer Action Network (PanCAN).^13-15^ Patients represented in Perthera’s RWE database were encouraged to undergo genomic profiling using a tumor biopsy sample at a CLIA-certified, CAP-accredited commercial laboratory with a comprehensive NGS testing panel. If prior NGS testing results were not available at the time of enrollment, Perthera optionally provided operational support to cancer care teams in coordinating the completion of genomic profiling at a commercial testing laboratory using formalin-fixed paraffin-embedded (FFPE) tissue samples gathered by either routine surgical resections, fine-needle aspirations, or core-needle biopsies. In general, biopsies acquired inside a year of testing were utilized for molecular profiling; however, archived biopsies were used in certain situations.

All patients represented in the analysis cohort of this study had NGS testing results available that were harmonized from commercial laboratory reports into a structured format and reviewed within the context of Perthera’s Virtual Molecular Tumor Board alongside each patient’s past medical and treatment history. Clinical features and treatment outcomes were manually curated from physician’s notes, pathology reports, radiology reports, and other medical records obtained via periodic records requests to patients’ treating institutions. The real-world study population included patients with pancreatic adenocarcinoma (*N* = 817) or pancreatic adenosquamous carcinoma (*N* = 14). Pancreatic neuroendocrine tumors, pancreatic acinar cell carcinoma, and other rare subtypes were excluded. Pathology information related to IPMN and cystic status were not taken into consideration due to limited data availability.

Documented sites of metastatic disease were retrospectively assessed through manual chart abstraction in 2094 patients with mPDAC that had NGS testing results from Perthera’s RWE database. From this population of 2094 patients enrolled between January 2012 through December 2022, we identified a total of 831 patients to include in the analysis cohort whose tumors were annotated as having first metastasized to either the lung (and not the liver) or the liver (and not the lung). Patients with evidence of metastatic disease presenting in both liver and lung within 180 days of the initial onset of metastatic disease were excluded from the analysis cohort. Patients with only non-lung/non-liver distant lesions (eg, peritoneum, bone, brain) at onset of metastatic disease were also excluded from the analysis cohort; however, the presence of other non-lung/non-liver lesions synchronous with onset of metastatic disease to either the liver or the lung were allowed. For patients diagnosed with non-metastatic disease at initial presentation, the earliest available progress note recorded by the treating oncologist that interpreted the patient’s disease as metastatic (eg, when initiating the first line of therapy for metastatic disease was considered an appropriate treatment plan) was the preferred source used to assess which sites were present at the onset of metastatic disease.

There are several weaknesses associated with the RWE research methods implemented here that are important to note. Chart abstracted information from pathology reports, imaging reports, and oncology progress notes were collected from a wide range of institutions in the US where standard imaging practices may vary and the biopsy-confirmed status of each distant lesion by pathology was not guaranteed. The use of liver-specific MRI to rule out multiple metastatic sites following detection of lung lesions via CT CAP scans could not be controlled due to the observational nature of this study. Repeat scans using different imaging platforms were taken into consideration when annotating distant lesions at metastatic presentation, particularly when CT AP scans do not evaluate the chest. In complex scenarios where the onset of metastatic disease was associated with lesions from a single major site but potential lesions at additional major sites were only noted as suspicious for metastatic disease, follow-up records describing the progression of these suspicious lesions in parallel with the initially confirmed lesions were taken into account on a case-by-case basis (eg, chest imaging results were unavailable or inconclusive when a surveillance CT scan of the abdomen and peritoneum first revealed liver lesions but the subsequent CT scan of the chest/abdomen/peritoneum showed lung lesions that were initially missed but likely present at onset of metastatic disease). For patients diagnosed with distant lesions identified within 180 days of initial presentation (eg, when surgery for locally advanced disease was aborted due to later scans and/or biopsies that revealed liver metastases), these lesions were considered metastatic at the time of initial diagnosis and any neoadjuvant therapy given was considered the first line of therapy for metastatic disease.

We retrospectively analyzed longitudinal clinical and treatment outcomes from the analysis cohort of 831 patients with PDAC whose tumors first metastasized to either the lung or the liver. Median overall survival (mOS) was primarily measured from the date of advanced diagnosis (ie, metastatic presentation) until death (uncensored event) or last encounter date (censored event). For subset analyses of resectable cases only, mOS was measured from the date of initial diagnosis of stage I-III disease (with successful resection) until death. The main analysis cohort combines patients who were initially diagnosed with either advanced or resectable disease in which all resectable cases had documented recurrence to the liver or lung. An important assumption of this study is that the prognosis of patients with recurrent disease is not substantially different from those who were initially diagnosed with advanced disease when analyzing mOS relative to the date of advanced diagnosis; however, the extent of disease burden (beyond stage) and performance status at advanced presentation represent potential confounders when analyzing a combined cohort as previously described.^15^ Median progression-free survival (mPFS) was evaluated from initiation of 1st-line (or 2nd-line) therapy for advanced disease until discontinuation due to disease progression (uncensored event) or until the most recent dose given if treatment was ongoing or discontinued for other reasons (censored event). Hazard ratios with 95% confidence intervals and *P*-values were computed via Cox regression between liver-specific and lung-specific subgroups. Differences in mPFS on either Gemcitabine/nab-paclitaxel or 5FU-based regimens (eg, FOLFIRINOX, FOLFOX, FOLFIRI, or 5-fluorouracil plus nal-Irinotecan) were assessed separately in both 1st-line and 2nd-line settings for advanced disease. Biological differences were evaluated by comparing frequencies of genomic alterations in the analysis cohort in patients with lung-specific versus liver-specific metastases using Fisher’s exact test without false discovery rate (FDR) correction.

## Results

We identified an analysis cohort of 831 patients with mPDAC whose tumors either metastasized distinctly to the lung (lung-only) [*n* = 142] or to the liver (liver-only) [*n* = 689] (Supplementary Figure S1). Clinical features as well as patient demographics were relatively balanced with exceptions relating to age and stage at diagnosis (Table 1).

**Table 1:** Clinical features and patient demographics.

	Analysis Cohort		1st-Line FOLFIRINOX/FOLFOX/FOLFIRI Cohort		2nd-Line FOLFIRINOX/FOLFOX/FOLFIRI Cohort		1st-Line Gemcitabine/nab-Paclitaxel Cohort		2nd-Line Gemcitabine/nab-Paclitaxel Cohort	
Patient Sex	Liver	Lung	Liver	Lung	Liver	Lung	Liver	Lung	Liver	Lung
Male	319 (46%)	82 (58%)	92 (44%)	31 (61%)	43 (41%)	14 (61%)	83 (47%)	16 (50%)	39 (43%)	11 (79%)
Female	370 (54%)	60 (42%)	119 (56%)	20 (39%)	62 (59%)	9 (39%)	92 (53%)	16 (50%)	51 (57%)	3 (21%)

	Analysis Cohort		1st-Line FOLFIRINOX/FOLFOX/FOLFIRI Cohort		2nd-Line FOLFIRINOX/FOLFOX/FOLFIRI Cohort		1st-Line Gemcitabine/nab-Paclitaxel Cohort		2nd-Line Gemcitabine/nab-Paclitaxel Cohort	
Age at Diagnosis	Liver	Lung	Liver	Lung	Liver	Lung	Liver	Lung	Liver	Lung
<65	403 (58%)	78 (55%)	156 (74%)	35 (69%)	63 (60%)	8 (35%)	85 (49%)	16 (50%)	64 (71%)	8 (57%)
≥ 65	286 (42%)	64 (45%)	55 (26%)	16 (31%)	42 (40%)	15 (65%)	90 (51%)	16 (50%)	26 (29%)	6 (43%)

	Analysis Cohort		1st-Line FOLFIRINOX/FOLFOX/FOLFIRI Cohort		2nd-Line FOLFIRINOX/FOLFOX/FOLFIRI Cohort		1st-Line Gemcitabine/nab-Paclitaxel Cohort		2nd-Line Gemcitabine/nab-Paclitaxel Cohort	
Stage at Diagnosis	Liver	Lung	Liver	Lung	Liver	Lung	Liver	Lung	Liver	Lung
IV	465 (67%)	32 (25%)	138 (65%)	20 (39%)	76 (72%)	4 (17%)	125 (71%)	11 (34%)	61 (68%)	8 (57%)
III	99 (14%)	38 (27%)	8 (4%)	4 (8%)	7 (7%)	2 (9%)	9 (5%)	0 (0%)	7 (8%)	2 (14%)
IIB	76 (11%)	38 (27%)	26 (12%)	12 (24%)	7 (7%)	6 (26%)	20 (11%)	9 (28%)	10 (11%)	1 (7%)
IIA	25 (4%)	10 (7%)	6 (3%)	2 (4%)	4 (4%)	1 (4%)	10 (6%)	3 (9%)	2 (2%)	1 (7%)
IB	5 (1%)	4 (3%)	1 (0%)	0 (0%)	0 (0%)	1 (4%)	1(1%)	1 (3%)	1 (1%)	0 (0%)
IA	0 (0%)	2 (1%)	0 (0%)	1 (2%)	0 (0%)	0 (0%)	0 (0%)	0 (0%)	0 (0%)	0 (0%)
Unavailable	19 (3%)	15 (10%)	32 (16%)	12 (23%)	11 (10%)	9 (40%)	10 (6%)	8 (26%)	9 (10%)	2 (15%)

	Analysis Cohort		1st-Line FOLFIRINOX/FOLFOX/FOLFIRI Cohort		2nd-Line FOLFIRINOX/FOLFOX/FOLFIRI Cohort		1st-Line Gemcitabine/nab-Paclitaxel Cohort		2nd-Line Gemcitabine/nab-Paclitaxel Cohort	
Background	Liver	Lung	Liver	Lung	Liver	Lung	Liver	Lung	Liver	Lung
White	384 (56%)	85 (60%)	134 (64%)	29 (57%)	67 (64%)	13 (57%)	103 (59%)	20 (62%)	62 (69%)	9 (64%)
Asian	25 (4%)	9 (6%)	8 (4%)	2 (4%)	6 (6%)	1 (4%)	8 (5%)	1 (3%)	2 (2%)	0 (0%)
Black	20 (3%)	3 (2%)	6 (3%)	1 (2%)	4 (4%)	0 (0%)	6 (3%)	0 (0%)	0 (0%)	0 (0%)
Hispanic	22 (3%)	3 (2%)	7 (3%)	2 (4%)	3 (3%)	1 (4%)	9 (5%)	1 (3%)	2 (2%)	0 (0%)
Ashkenazi	16 (2%)	1 (1%)	6 (3%)	1 (2%)	1 (1%)	0 (0%)	4 (2%)	0 (0%)	1 (1%)	0 (0%)
Other	11 (2%)	3 (2%)	3 (1%)	2 (4%)	2 (2%)	1 (4%)	2 (1%)	1 (3%)	1 (1%)	0 (0%)
Unavailable	211 (30%)	38 (27%)	47 (22%)	14 (27%)	22 (20%)	7 (31%)	43 (25%)	9 (29%)	22 (25%)	5 (36%)

### Metastasis to the lung vs the liver was prognostically favorable in a real-world mPDAC cohort

**Figure 1.**
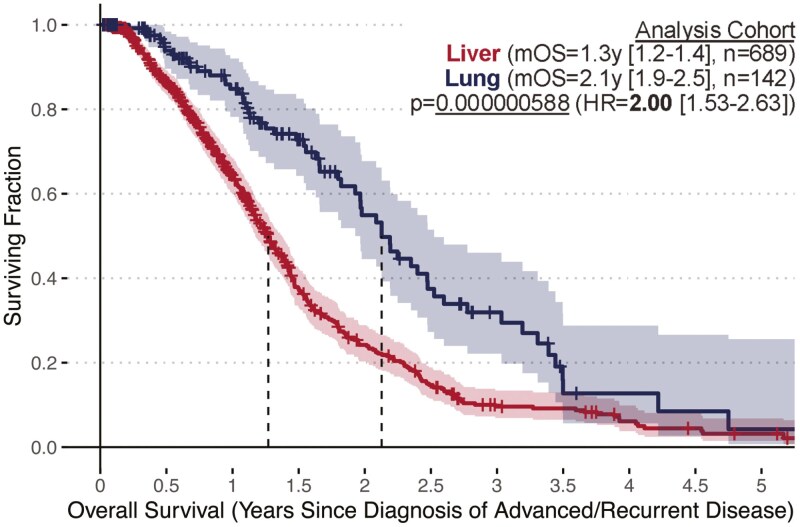
Overall survival outcomes within the Analysis Cohort compared between patients with lung-only vs. liver-only distant lesions at onset of metastatic disease while using the advanced diagnosis date as the start of the OS interval (note: any stage was eligible at initial diagnosis for the Analysis Cohort).

**Figure 2.**
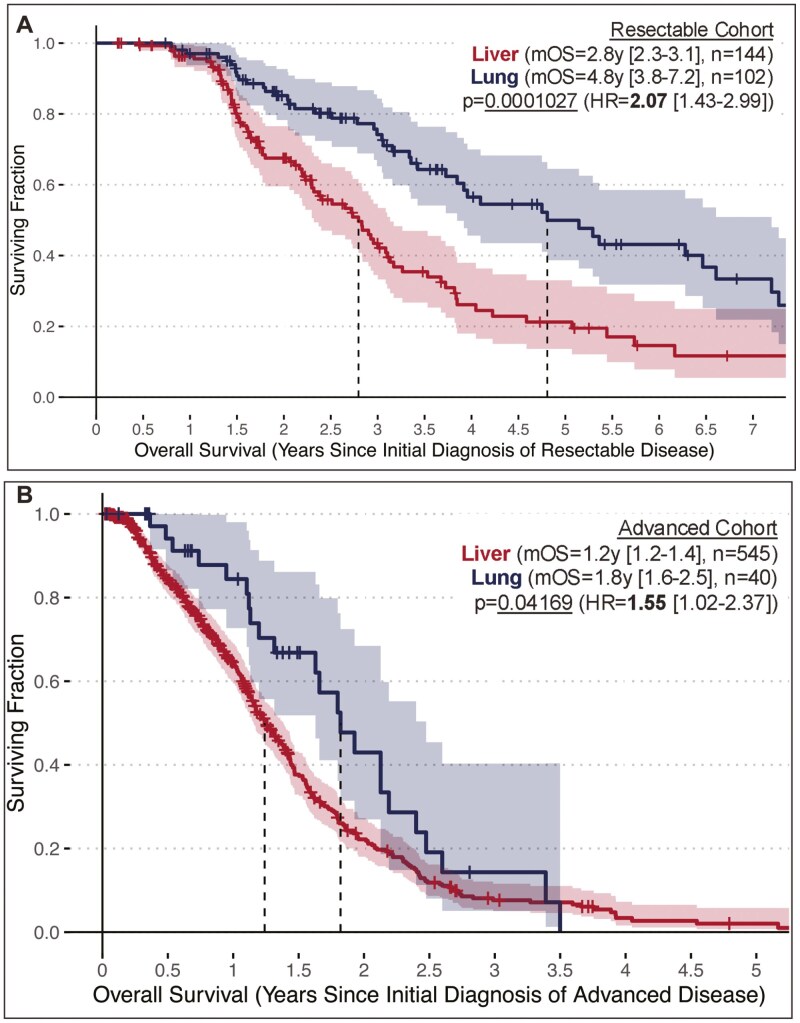
(A) Overall survival outcomes for the Resectable Cohort, a subset of the Analysis Cohort with earlier stage disease that resulted in successful resection, analyzed between lung-only vs liver-only cases using Cox regression while using date of initial diagnosis as the start of the OS interval. (B) Overall survival analysis between lung-only vs liver-only cases in the Advanced Cohort based on the initial diagnosis date, which aligns with the advanced diagnosis date since these subjects were not considered eligible for curative surgery.

To understand potential prognostic differences between lung-only and liver-only subsets of mPDAC, overall survival outcomes were analyzed relative to date of advanced diagnosis across the entire analysis cohort irrespective of initial staging. We found mOS following diagnosis of advanced/recurrent/metastatic disease was significantly longer in the lung-only cohort (2.1y [1.9-2.5], *n* = 142) compared to the liver-only cohort (1.3y [1.2-1.4], *n* = 689) (*P* = 0.000000588, HR = 2.00 [1.53-2.63]) (Figure 1). We also analyzed overall survival in the subset of 246 patients with resectable disease who later presented with metastatic recurrence and the subset of 585 patients who initially presented with advanced disease. Within the resectable cohort, mOS relative to the date of initial diagnosis was significantly longer in the lung-only subset (mOS = 4.8y [3.8-7.2], *n* = 102) vs the liver-only subset (mOS = 2.8y [2.3-3.1], *n* = 144) (*P* = 0.0001027, HR = 2.07 [1.43-2.99], Figure 2A). Within the advanced cohort, mOS relative to the date of advanced presentation remained significantly longer in the lung-only subset (mOS = 1.8y [1.6-2.5], *n* = 40) compared to the liver-only subset (mOS = 1.2y [1.2-1.4], *n* = 545) (*P* = 0.04169, HR = 1.55 [1.02-2.37], Figure 2B).

### Overall survival differences between lung-only and liver-only metastasizing PDAC are observed in all standard 1st-line therapies

**Figure 3.**
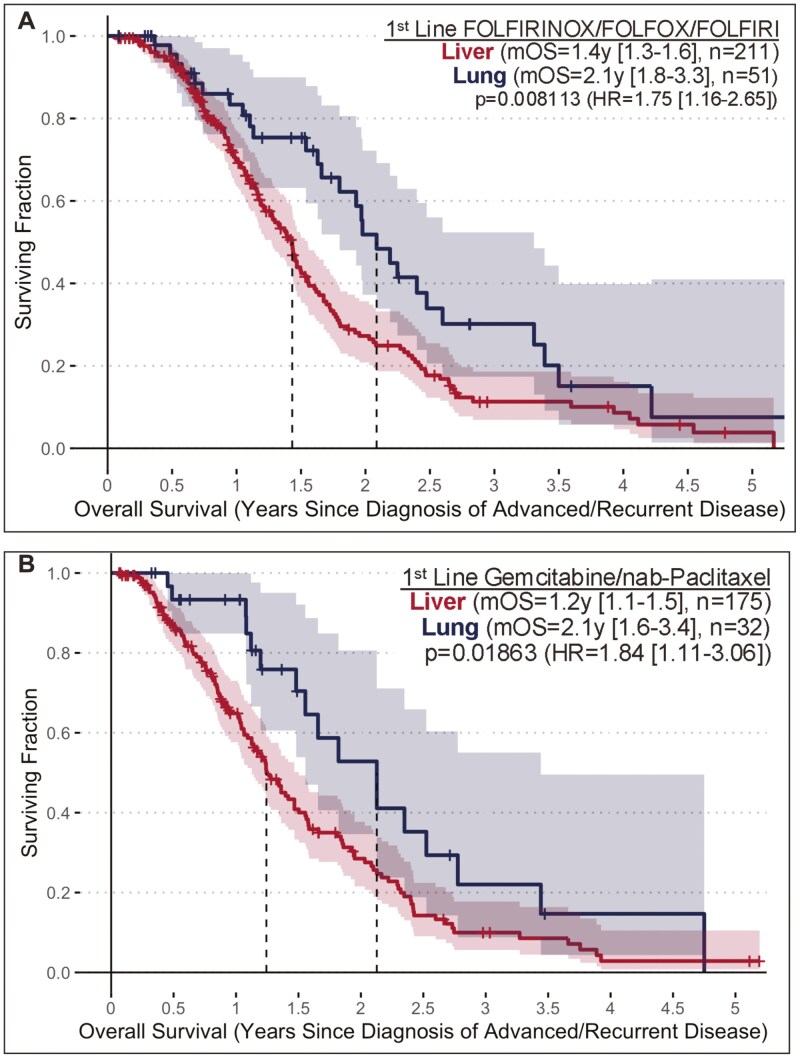
(A) Median OS relative to diagnosis of advanced disease for the subset receiving 1st-line 5FU-based SOC in lung-only vs. liver-only subsets of the mPDAC Analysis Cohort. (B) Median OS relative to diagnosis of advanced disease for the subset receiving 1^st^-line Gemcitabine/nab-Paclitaxel SOC in lung-only vs. liver-only subsets of the mPDAC Analysis Cohort.

Median overall survival was analyzed for patients on 1st-line FOLFIRINOX/FOLFOX/FOLFIRI (5FU-based) or gemcitabine/nab-paclitaxel standard-of-care (SOC) therapies and compared between those that developed lung-only and liver-only metastases. For patients receiving 1st-line 5FU-based SOC therapies, there was a significant difference in mOS between patients that developed liver-only metastasis (mOS = 1.4y [1.3-1.6], *n* = 211) and those that developed lung-only metastasis (mOS = 2.1y [1.8-3.3, *n* = 51) (*P* = 0.008113, HR = 1.75 [1.16-2.65]) (Figure 3A). Similarly, for patients receiving SOC gemcitabine/nab-paclitaxel therapy in the 1st-line setting, there was a significant difference in mOS between those that developed liver-only metastasis (mOS = 1.2y [1.1-1.5], *n* = 175) and those that developed lung-only metastasis (mOS = 2.1y [1.6-3.4, *n* = 32) (*P* = 0.01863, HR = 1.84 [1.11-3.06]) (Figure 3B).

**Figure 4.**
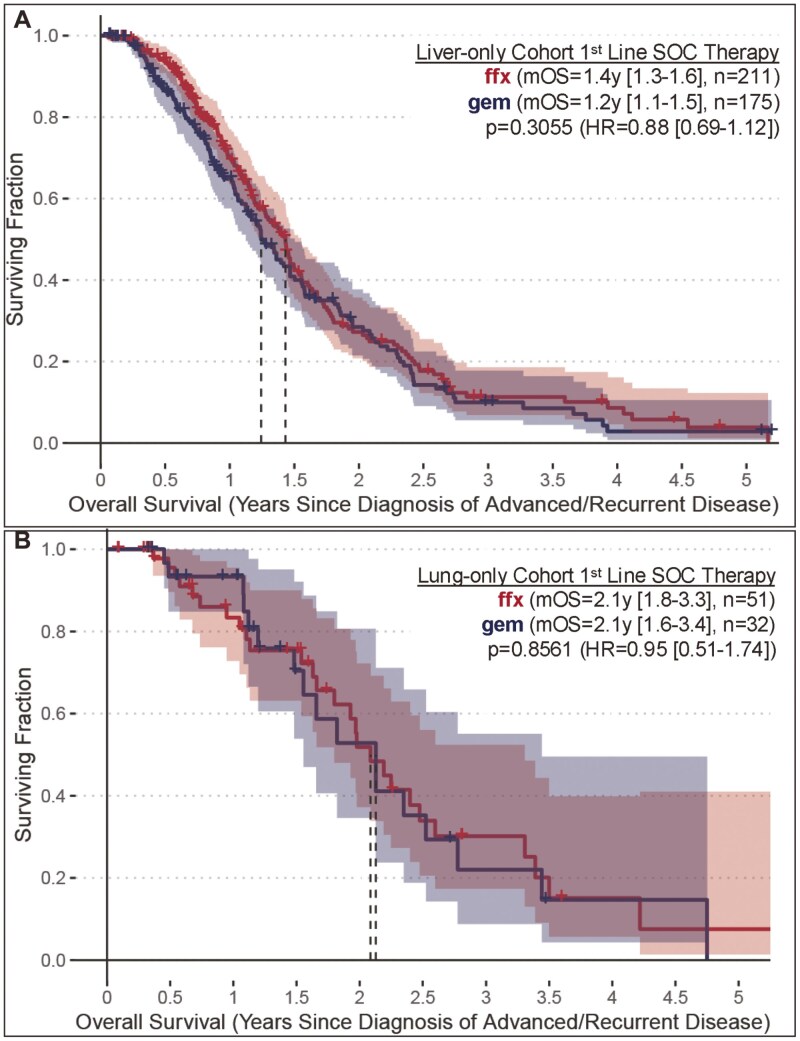
(A) Median OS relative to diagnosis of advanced disease for the subset receiving 1st-line 5FU-based SOC vs. 1st-line Gemcitabine/nab-Paclitaxel SOC in the liver-only subset of the mPDAC Analysis Cohort. (B) Median OS relative to diagnosis of advanced disease for the subset receiving 1st-line 5FU-based SOC vs. 1st-line Gemcitabine/nab-Paclitaxel SOC in the lung-only subset of the mPDAC Analysis Cohort.

Within the liver-only cohort, no statistical difference in mOS was found when comparing patients that received 1st-line 5FU-based therapy to those receiving 1st-line gemcitabine/nab-Paclitaxel therapy (*P* = .3055, HR = 0.88 [0.69-1.12]) (Figure 4A). Similarly, within the lung-only cohort, no statistical difference in mOS was found when comparing patients that received 1st-line 5FU-based therapy to those receiving 1st-line gemcitabine/nab-paclitaxel therapy (*P* = 0.8561, HR = 0.95 [0.51-1.74]) (Figure 4B).

### 1st- and 2nd-line SOC 5FU-based and gemcitabine/nab-paclitaxel-based therapies demonstrate some differences in progression-free survival in lung- vs liver-metastasizing PDAC

Median progression-free survival was analyzed for patients on 1st- and 2nd-line 5FU- based or gemcitabine/nab-paclitaxel SOC therapies and compared between those that developed lung-only and liver-only metastases. For patients receiving 1st-line 5FU-based SOC therapies, there was no significant difference in mPFS between patients that developed liver-only metastasis (mPFS = 8.9m [7.1-10.5], *n* = 211) and those that developed lung-only metastasis (mPFS = 10.3m [8.2-N/R, *n* = 51) (*P* = 0.07629, HR = 1.52 [0.96-2.42]) (Supplementary Figure 2A). Likewise, for patients receiving SOC gemcitabine/nab-paclitaxel therapy in the 1st-line setting, there was no significant difference in mPFS between those that developed liver-only metastasis (mPFS = 6.1m [5.6-7.6], *n* = 175) and those that developed lung-only metastasis (mPFS = 7.9m [6.1-N/R, *n* = 32) (*P* = 0.09844, HR = 1.52 [0.9-2.50]) (Supplementary Figure S2B).

For patients receiving 2nd-line 5FU-based SOC treatment regimens, no significant difference was found in mPFS between patients with liver-only metastasis (mPFS = 4.3m [3.8-5.6], *n* = 105) and patients with lung-only metastasis (mPFS = 5.1m [3.1-N/R], *n* = 23) (*P* = 0.6800, HR1.13 [0.64-2.00]) (Supplementary Figure S3A). However, patients receiving gemcitabine/nab-paclitaxel in the second-line setting demonstrated a significant difference in mPFS between those that developed liver-only metastasis (mPFS = 4.1m [2.9-4.9], *n* = 90) and those that developed lung-only metastasis (mPFS = 9.3m [6.3-N/R, *n* = 14) (*P* = 0.01337, HR = 2.76 [1.23-6.18] (Supplementary Figure S3B).

### Unique molecular signatures of liver-only metastasizing PDAC and lung-only metastasizing PDAC

**Figure 5.**
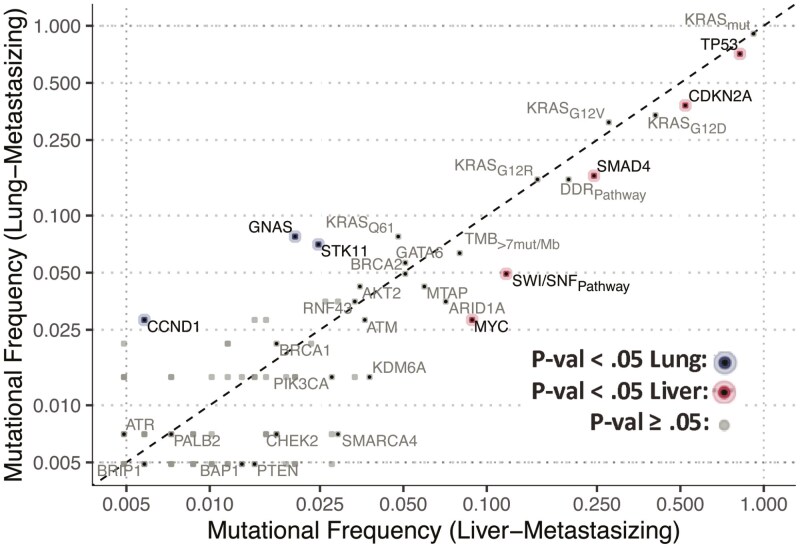
Genomic alteration frequencies in lung- vs. liver-metastasizing PDAC.

Molecular profiling of the pancreatic tumors demonstrated significant differences in the frequencies of several known PDAC mutations between patients with lung- and liver-only metastasis. PDAC tumors with liver-only metastases were modestly enriched (unadjusted *P* < 0.05) for: *TP53* mutations (81.4% in liver vs 69.2% in lung), *MYC* amplifications (8.6% in liver vs 3.0% in lung), inactivating *CDK2NA* alterations (51.5% in liver vs 39.1% in lung), inactivating *SMAD* alterations (24.4% in liver vs 16.3% in lung), and mutations in the *SWI/SNF* pathway (11.8% in liver vs 4.9% in lung) (Figure 5; Table 2). PDAC tumors with lung-only metastases were modestly enriched (unadjusted *P* < 0.05) for: *STK11* mutations (2.5% in liver vs 7.0% in lung), *CCND1* amplifications (0.6% in liver vs 2.8% in lung), and *GNAS* alterations (2.0% in liver vs 7.8% in lung) (Figure 5; Table 2). No significant differences were noted in either *KRAS* mutations or specific isoforms between lung-only and liver-only metastasis (Figure 5; Table 2).

**Table 2:** Genomic alteration frequencies in lung- vs. liver-metastasizing PDAC.

Enriched Gene	Enrichment Trend	Liver Frequency		Lung Frequency
*TP53*	Mutations	**82.0%**	>	71.3%
*MYC*	Amplifications	**8.9%**	>	2.8%
*CDK2NA*	Mutations/Loss	**52.1%**	>	38.0%
*SMAD4*	Mutations/Loss	**24.4%**	>	16.2%
*SWI/SNF*	Mutations/Loss	**11.8%**	>	4.9%
*STK11*	Mutations	2.5%	<	**7.0%**
*CCND1*	Amplifications	0.58%	<	**2.82%**
*GNAS*	Alterations	2.0%	<	**7.8%**
*KRAS [ANY]*	Mutations	91.9	~	90.9
*KRAS G12D*	G12D Variant	40.6	~	33.8
*KRAS G12V*	G12V Variant	27.6	~	31.0
*KRAS G12R*	G12R Variant	15.2	~	15.5
*KRAS Q61*	Q61 Variant	4.8	~	7.8
*KRAS G12C*	G12C Variant	2.0	~	0.0
*KRAS ETC*	Other Alterations	1.6	~	2.8
*ARID1A*	Mutations/Loss	7.1	~	3.5
*MTAP*	Mutations/Loss	6.0	~	4.2
*GATA6*	Mutations/Loss	5.1	~	5.6
*BRCA2*	Mutations/Loss	5.1	~	4.9
*AKT2*	Mutations/Amplifications	3.5	~	4.2
*ATM*	Mutations/Loss	3.6	~	2.8
*KDM6A*	Mutations/Loss	3.8	~	1.4
*RNF43*	Mutations/Loss	3.3	~	3.5
*CCNE1*	Amplifications	3.3	~	1.4
*KMT2D*	Mutations/Loss	2.9	~	3.5
*CDK6*	Amplifications	2.6	~	3.5
*SMARCA*	Mutations/Loss	2.9	~	0.7
*PIK3CA*	Mutations/Amplifications	2.8	~	1.4
*DNMT3A*	Mutations/Loss	2.8	~	0.7
*MAP2K4*	Mutations/Amplifications	2.8	~	0.0
*ERBB2*	Mutations/Amplifications	2.3	~	2.1
*KDM5A*	Mutations/Loss	2.0	~	1.4
*LRP1B*	Mutations/Loss	2.0	~	1.4
*RB1*	Mutations/Loss	2.0	~	1.4
*SF3B1*	Mutations/Loss	2.0	~	1.4
*BRCA1*	Mutations/Loss	1.7	~	2.1
*U2AF1*	Mutations/Loss	1.9	~	1.4
*ZNF703*	Mutations/Loss	2.0	~	0.0
*KMT2C*	Mutations/Loss	1.5	~	2.8
*FGFR1*	Mutations/Amplifications	1.9	~	0.0
*CCND2*	Amplifications	1.7	~	0.7
*CHEK2*	Mutations/Loss	1.7	~	0.7

## Discussion

The aim of this study was to understand survival outcomes, impact of treatment and molecular phenotypes of site-specific mPDAC. Our data confirmed prior clinical findings that lung-only metastasis is a positive prognostic factor for metastatic PDAC.^7,8,12,16^ Patients with lung-only mPDAC demonstrated a clinically and statistically significantly longer mOS when compared to patients with liver-only metastatic disease. Similar trends were also observed in our mPFS results. Our genomic analysis of the PDAC tumors which metastasized either to the liver-only or the lung-only revealed unique molecular signatures. The significant difference in the mutational profile between metastatic sites suggests that lung-only mPDAC tumors lack inherent genomic features found in liver-only metastases, which may contribute towards a differing biology and positive prognostic value for lung-only mPDAC.

Our results reinforce prior clinical findings that patients with lung-only mPDAC exhibit a more positive prognosis relative to mPDAC patients with liver-only metastasis. Prognosis in lung-only mPDAC patients is significantly longer than liver-only mPDAC; however, the response to chemotherapy does not explain these differences. Statistically significant differences in mOS were observed between liver-only and lung-only cohorts regardless of the type of frontline SOC backbone chemotherapy given. Furthermore, within both the liver-only and lung-only mPDAC cohorts, there was no statistical difference in mOS between patients that received 1st-line 5FU-based or gemcitabine/nab-paclitaxel SOC therapy. Although in most cases comparative mPFS in both the 1st- and 2nd-line settings did not show statistically significant differences between liver-only and lung-only patients, a general trend towards more favorable outcomes in lung-only patients is observed. Future analyses with larger samples sizes may further expound on our mPFS data and reveal where in a patient’s treatment history (ie, line of therapy) the survival benefits of lung-only disease take effect. Interestingly, our analysis did reveal a statistically significant longer mPFS for lung-only patients receiving 2nd-line gemcitabine/nab-paclitaxel relative to liver-only patients receiving the same treatment, a finding that supports our mOS observations. The longer mPFS trends in lung-only mPDAC cohorts support our hypothesis of lung-only patients having a more favorable diagnosis. Collectively, these results point to factors other than the impact of treatment on the prognosis differences between these two cohorts, namely the specific site of metastasis.

Liver-only mPDAC was modestly enriched for *TP53* mutations, *MYC* amplifications, *CDK2NA* inactivating alterations, mutations in the *SWI/SNF* pathway, and *SMAD4* inactivating alterations, which impart a more aggressive disease characteristic to the tumor relative to lung-only mPDAC. *MYC* amplifications are associated with poorer prognosis in various types of cancer and are suggested to affect the progression of exceedingly aggressive forms of PDAC.^5,17-19^*CDK2NA* inactivation has been found in aggressive meningiomas and is indicated as one of the drivers of poor prognosis in PDAC.^20-22^ Vitellius et al. suggest that the lack of alterations in *CDK2NA* and *SMAD4* tumor suppressor genes in patients with lung-only mPDAC are linked with better overall survival, a finding in line with our study.^23^ Additionally, increased tumor aggressiveness due to *TP53* alterations has been documented in several cancers, including colorectal cancer, breast cancer, and PDAC, with *TP53* proposed as a possible biomarker for recurrence and metastatic disease in prostate, endometrial, and epithelial ovarian cancers.^22,24-29^ Within the *SWI/SNF* pathway, two protein complex subunits, BAF and pBAF, had significantly more genetic alterations in the liver-only cohort. Several studies implicate loss-of-function abnormalities in this pathway as a driver of tumorigenesis, with nearly 20%-25% of all cancers bearing genetic alterations in the chromatin-remodeling complexes of this pathway.^30-33^

Our study reveals novel and different genetic alterations enriched in lung-only mPDAC relative to liver-only disease: *STK11* mutations, *CCND1* amplifications, and *GNAS* alterations. Our data showed that while exhibiting a longer mOS than liver-only mPDAC, lung-only mPDAC is still lethal. *STK11/LKB1* loss of function alterations enhance cell proliferation and promote cancer cell growth, motility, and invasion, thus enhancing metastatic potential.^34,35^ However, the other two genetic alterations enriched in lung-only mPDAC do not unquestionably bestow an aggressive tumor characteristic; a finding that differs from that of liver-only mPDAC. Studies exploring *CCND1* in various cancer types report conflicting information regarding the prognostic value of this mutation, varying from reduced survival to increased survival, with some reporting no association at all.^36-45^ Furthermore, *GNAS* alterations have been suggested to dampen the aggressiveness of some PDAC tumors. The mutant *GNAS* oncogene can suppress cancer cell growth in some human pancreatic cancer cells by antagonizing the *KRAS* pathway, thus limiting the aggressiveness of the tumor.^46,47^ Interestingly, GNAS has been found to be a common genetic mutation in intraductal papillary mucinous neoplasms (IPMN), being identified in 40%-70% of lesions, and thus may be a genetic driver of IPMN progression to malignancy.^48,49^ Furthermore, IPMNs have been shown to display a recurrence pattern with higher propensity of metastasis to the lung.^50^ Our findings on GNAS alterations being present significantly more in lung-only patients relative to liver-only patients are in line with prior reports on potential relationships between GNAS, IPMN, and lung metastasis; however, a dataset richer in IPMN annotations would be needed to solidify any conclusions.

This study has shown that mPDAC is a heterogenous cancer at the molecular level with varying clinical outcomes regarding prognosis that do not seem to be related to current chemotherapeutic responses. Organotropic metastatic tumor diversity seems to be mirrored at the molecular level suggesting benefits of molecular categorizations of mPDAC. Furthermore, since cancer origination and progression results from complex interactions between tumors and their microenvironment, integrating our data with future immunohistochemical, transcriptomic and proteomic data can help pave the way in identifying patients at higher risk of metastasis and therefore poorer prognoses. Increased genomic insight into drivers of organ-specific metastatic spread is not unique to mPDAC. Retrospective data from Michl et al. suggests that *MAPK* pathway mutations that enhance the activity of the *Wnt/β-catenin* pathway are involved in the development of lung metastasis in metastatic colorectal cancer (mCRC), while high expression of CD133 correlates with liver metastasis in mCRC.^51^ Thus, our data can be utilized in the design of future clinical trials employing comprehensive gene expression profiling in a precision medicine-based approach to treat PDAC patients.

Differences in survival outcomes based on site specific metastases suggests that current approaches to the standard management practices of patients with oligometastatic PDAC should be updated. Prior investigations have already demonstrated that oligometastatic pancreatic cancer may be regarded as its own unique stage of disease, deserving its own evaluation.^52^ Given the better prognosis for patients with lung-only oligometastatic PDAC, a more aggressive approach involving surgical resection of residual oligometastatic disease following upfront chemotherapy may provide further benefit than just chemotherapy alone to this specific patient population. Identification of prognostic markers to predict the possibility of metastasis to the liver or lung and earlier detection of potentially resectable tumor can aid in the multidisciplinary management of patients based on their individual prognosis. Our data supports sites of metastasis as being one of the most important prognostic variables and should be considered as a stratification factor for clinical trials and included in clinical decision making. Large multicentric prospective clinical trials focused on oligometastatic PDAC patients are needed to confirm the potential benefit of surgery and other aggressive muti-modal therapeutic approaches. Additionally, our data informs clinicians of the prognostic value of site-specific metastases in mPDAC, which should be conveyed to patients as this may influence disease management.

Our study includes a large sample size for each metastatic site-specific cohort, provides insights into the differences of metastases based on genomics, and stratifies survival outcomes based on resectable versus non-resectable disease. However, our study has several limitations. While our work elucidated the unique genomic features of lung-only versus liver-only mPDAC, it did not fully explore specific differences in signaling pathways and biology such as the tumor microenvironment (TME) composition, methylation patterns, and the immune milieu between PDAC metastasis at these two sites. Whole transcriptome analysis of site-specific metastasis may provide deeper and novel insight into the pathways, which may impact both site of metastasis and clinical course of the disease. Additionally, earlier pre-clinical investigations in various advanced cancer types have shown differences in the tumor and its TME to be dependent on the metastatic site.^53^ Distinct cancer-associated fibroblasts (CAF), myeloid derived suppressor cells, macrophages, neutrophils, cytokines, neutrophil-derived factors, and other immune cells have all been identified as critical regulators of the extra-cellular matrix (ECM) and have been shown to influence metastasis.^54-58^ More specifically with pancreatic cancer, pre-clinical models note site-specific differences in key immunoregulatory pathways that distinguish hepatic and pulmonary mPDAC.^59^ Investigations broadening our understanding into factors that stimulate response to therapy in lung-only mPDAC patients can hopefully equip us with methods to mimic similar effects in liver-only mPDAC patients.

While our results suggest a possible predictive value of knowing the genomic alterations enriched in primary tumors towards evaluating patients at risk for developing metastasis at specific sites, further exploration with matched primary and metastatic samples from the same patient would strengthen this assessment. This retrospective real-world study was considered exploratory in nature and designed to understand the molecular underpinnings of mPDAC tumors that preferentially migrate to and develop in the lung in contrast to the liver, which is more typical. It is important to note that NGS testing results generated from primary tumor specimens were allowed. This may result in decreased sensitivity to detect genomic alterations that were absent in the primary tumor and only acquired after spreading to distant lesions. However, a previous study leveraging Perthera’s RWE database to examine genomic differences based on the sites of tumors specimens submitted for NGS profiling found that only a small number of biomarkers were trending towards enrichment in liver samples (more *MYC* amplifications) or lung samples (more *STK11* alterations) when compared to primary pancreatic samples.^5^ In contrast to the previous study, which rationalized that primary tumor specimens were suitable for identifying potentially actionable biomarkers in mPDAC, this study aims to better understand the clinical and molecular characteristics of tumors that have a propensity to spread to either the liver or the lung.

## Conclusion

Patients with lung-only mPDAC exhibit a more positive prognosis relative to mPDAC patients with liver-only metastasis. Patients with lung-only mPDAC demonstrated a statistically significantly longer mOS when compared to patients with liver-only metastatic disease. These survival differences seem to be related to factors outside of response to chemotherapy, particularly the site of metastasis. Genomic analysis of the PDAC tumors which metastasized either to the liver-only or the lung-only revealed unique molecular signatures. The mutational frequency differences between patients with liver-only and lung-only metastasis warrants a deeper investigation into the molecular drivers and associated pathways of site-specific metastases. Future studies supplementing our results with proteomic data on the TME and epigenetics at each site will broaden our understanding of the biologic drivers of disease metastasis and aggressiveness and contribute to the growing dataset of biomarkers, helping to identify unique subgroups amenable to immune therapy in advanced PDAC.

## Supplementary Material

oyaf007_suppl_Supplementary_Figures_1

oyaf007_suppl_Supplementary_Figures_2

oyaf007_suppl_Supplementary_Figures_3

## Data Availability

Any data underlying this article that is not available in the article itself will be shared on reasonable request to the corresponding author.
