# Supplementary material for: Clinical outcomes and molecular characteristics of lung-only and liver-only metastatic pancreatic cancer: results from a real-world evidence database
Source: Oncologist. 2025 Mar 13;30(3):oyaf007. doi: 10.1093/oncolo/oyaf007 (PMC11904785; doi:10.1093/oncolo/oyaf007)
Supplement: oyaf007_suppl_Supplementary_Figures_1 [file oyaf007_suppl_supplementary_figures_1.pdf]

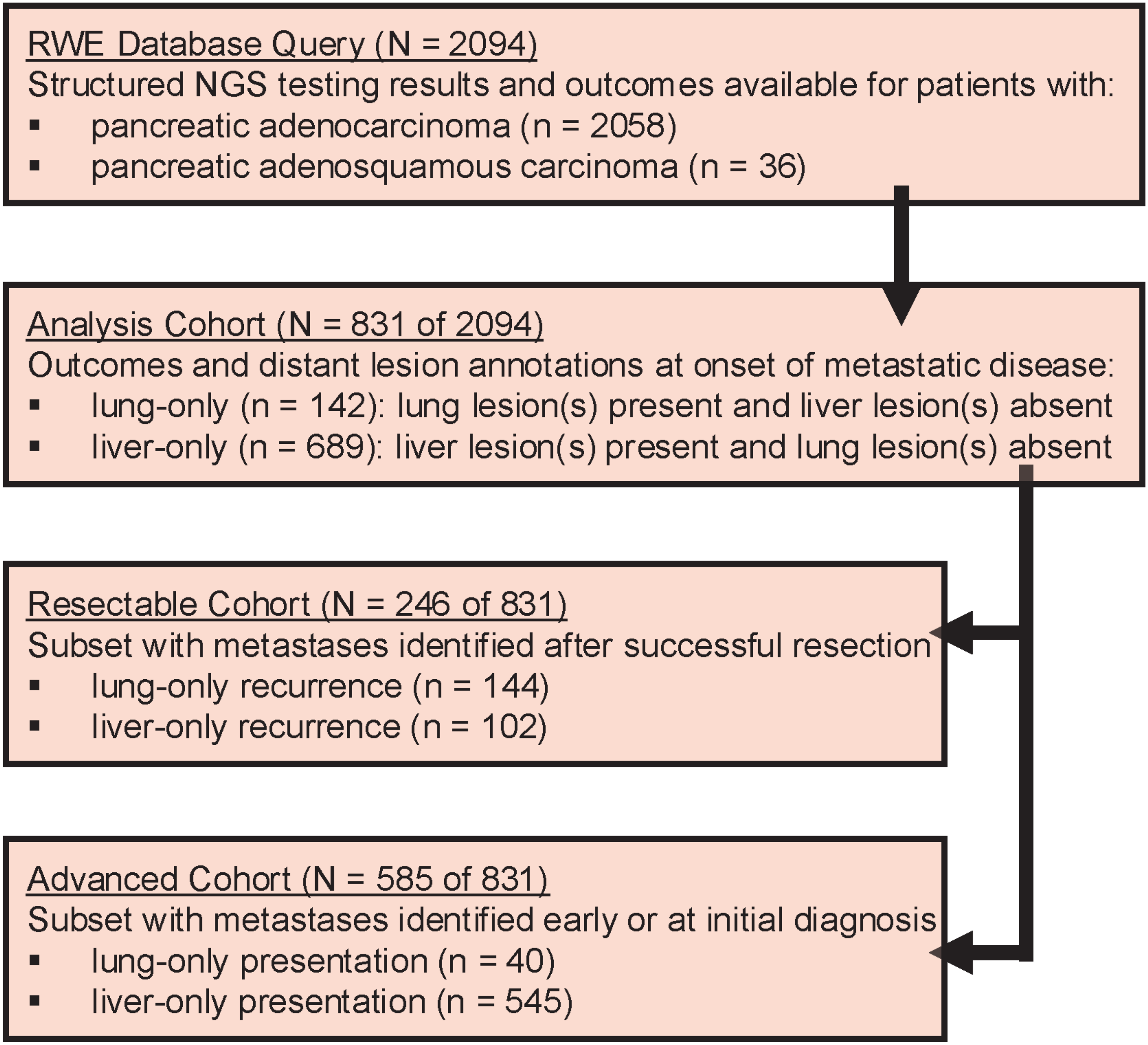
RWE Database Query (N = 2094)
Structured NGS testing results and outcomes available for patients with:
▪ pancreatic adenocarcinoma (n = 2058)
▪ pancreatic adenosquamous carcinoma (n = 36)
Analysis Cohort (N = 831 of 2094)
Outcomes and distant lesion annotations at onset of metastatic disease:
▪ lung-only (n = 142): lung lesion(s) present and liver lesion(s) absent
▪ liver-only (n = 689): liver lesion(s) present and lung lesion(s) absent
Resectable Cohort (N = 246 of 831)
Subset with metastases identified after successful resection
▪ lung-only recurrence (n = 144)
▪ liver-only recurrence (n = 102)
Advanced Cohort (N = 585 of 831)
Subset with metastases identified early or at initial diagnosis
▪ lung-only presentation (n = 40)
▪ liver-only presentation (n = 545)
