# Supplementary figures and images for: Clinical outcomes and molecular characteristics of lung-only and liver-only metastatic pancreatic cancer: results from a real-world evidence database
Source: Oncologist. 2025 Mar 13;30(3):oyaf007. doi: 10.1093/oncolo/oyaf007 (PMC11904785; doi:10.1093/oncolo/oyaf007)

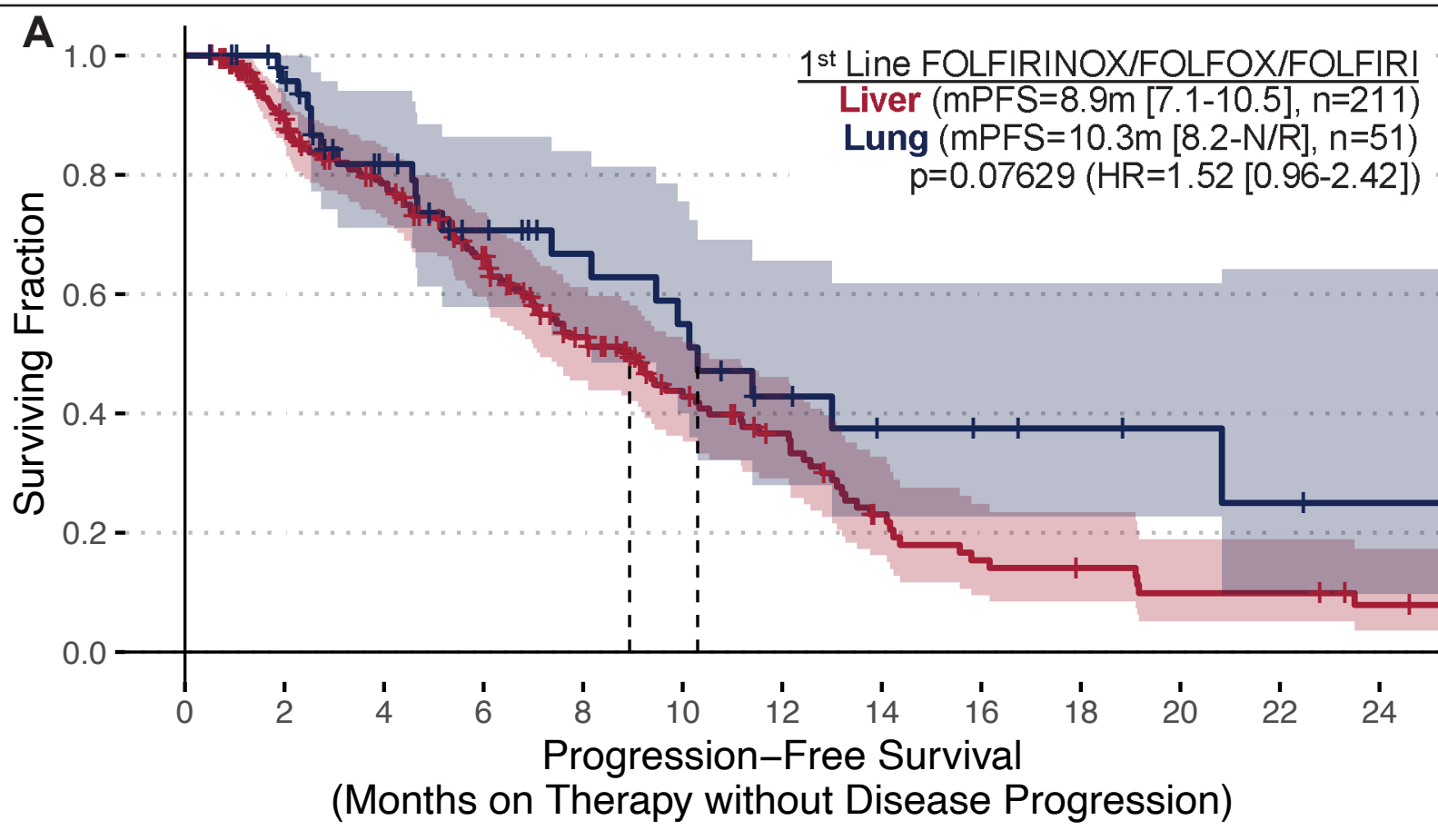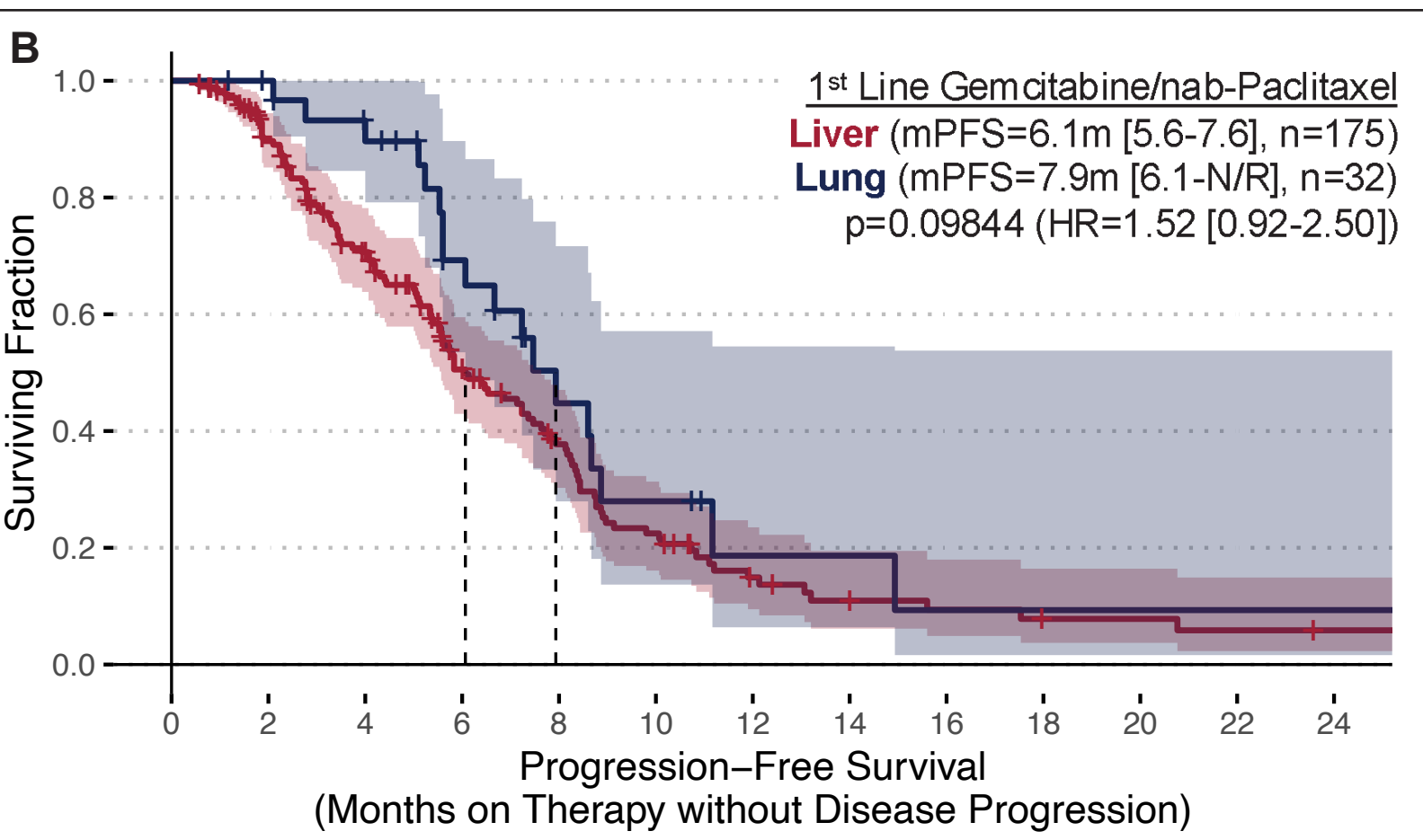

Supplement: oyaf007_suppl_Supplementary_Figures_2 [file oyaf007_suppl_supplementary_figures_2.pdf]

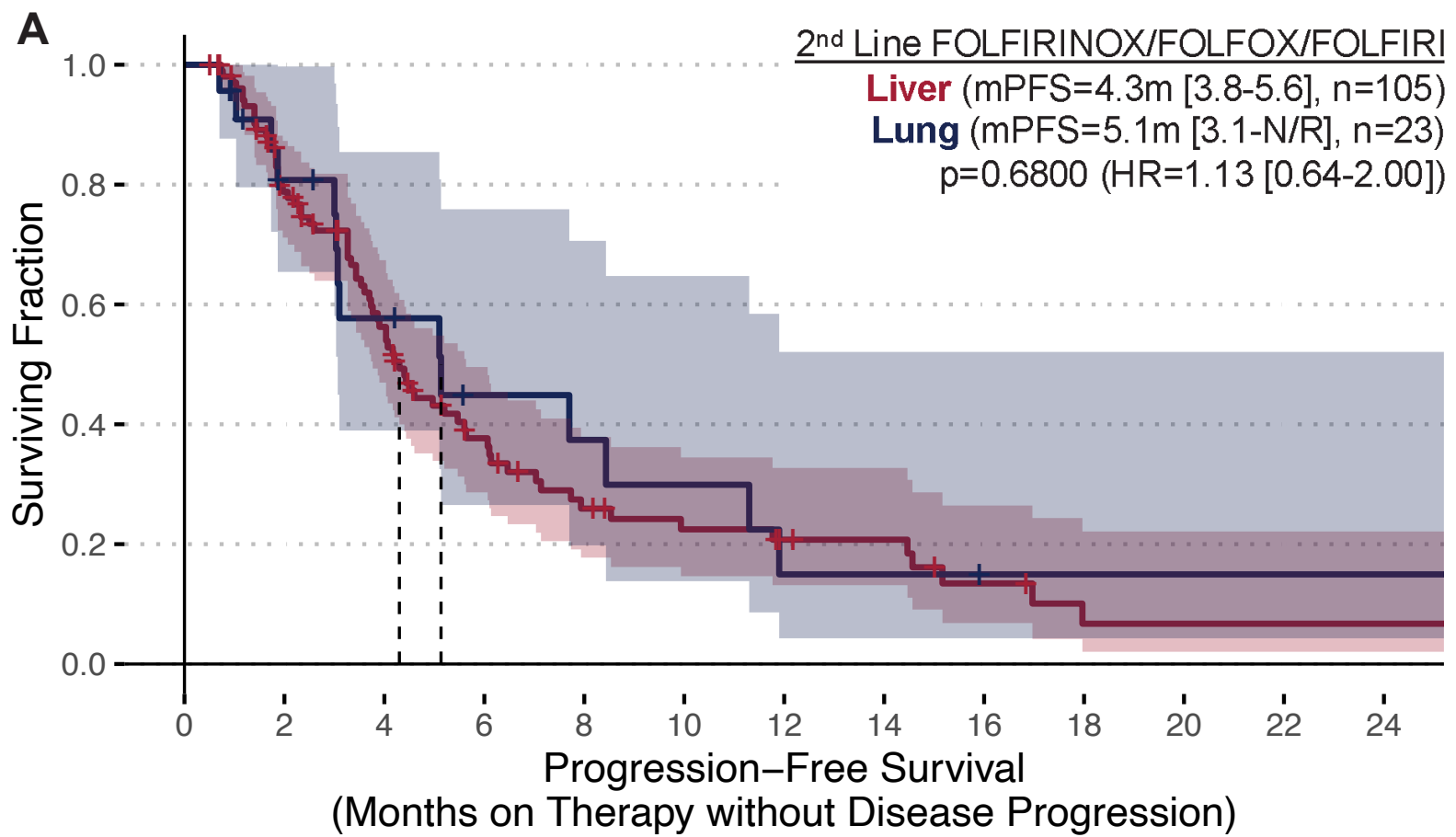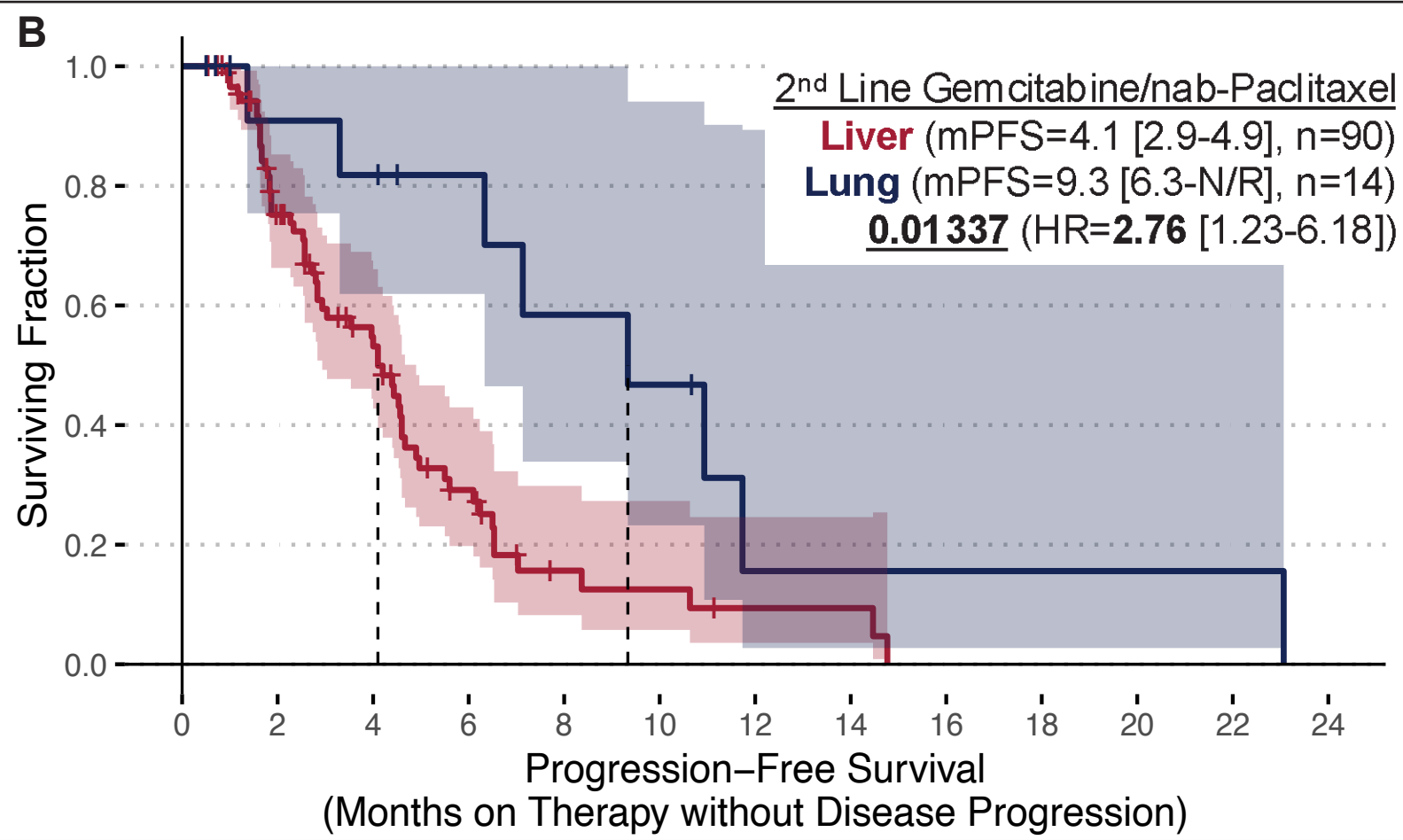

Supplement: oyaf007_suppl_Supplementary_Figures_3 [file oyaf007_suppl_supplementary_figures_3.pdf]
